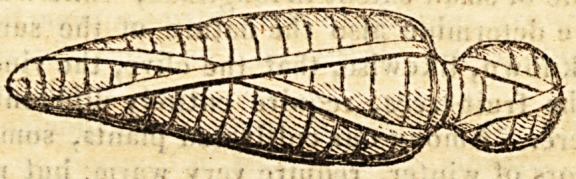# An Account of the Opening of an Egyptian Mummy

**Published:** 1821-10

**Authors:** 


### *An Account of the Opening of an Egyptian Mummy.* By the Editor.

THE mummy, the appearances of which I am about to detail, proved to be that of a female, and was presented to me by Sir Archibald Edmonston, Bart. who had purchased it at Thebes, in March 1819. It came out of one of the numerous catacombs in a perforated rock, and was found in an ornamented case made of sycamore wood, covered with painted hieroglyphics.

I proceeded to open it on the 29th of August last, in the presence of several friends, among whom were two medical gentlemen, Dr. Hutchinson and Mr. Alexander Copland Hutchison, who kindly assisted me in the operation.

It is not my purpose, on the present occasion, to enter into an antiquarian discussion on the subject of mummies, as they have been found in Egypt: such a course would be foreign to the object of this Journal; and it would, moreover, be presumptuous in me to advance any conjecture after the very full and satisfactory investigation of the subject, for which we are indebted to two very recent writers, M. Jomard and Baron Larrey. In the third and fourth volumes of the *Commentationes Societatis Regiæ Gottingensis*, the reader will also find much curious matter on the subject of Egyptian mummies, by Professor Hayne, as far as regards their antiquity; and by J. F. Gmelin, on the score of their physical properties.

An elaborate article, under the head *Momie*, will also be found in the *Dictionnaire des Sciences Medicales*, where the writer endeavours, by philologically scrutinizing the name "mummy," to ascertain its derivation: some authors, according to him, pretending that the name is derived from two Cophtic words, signifying *salt* and *death;* while others trace it to the monosyllable *mum*, the representative for *wax*. The probability, however, is, that the name mummy, in Latin, *mumia*, was given to the dry preparations in question, from the circumstance of the principal ingredient, originally employed in preserving them, being called *mummi-aï*, a species of mineral pitch, or *petroleum*, (not the *pisasphaltus*,) found in Arabia.

It distils from fissures in rocks, is of a bright-yellowish colour, and of the greatest purity. Its scarcity renders it extremely dear, so that it is often sold at a very high price; and Sir Gore Ouseley, whose practical knowledge of Oriental customs and language is very extensive, informs me that the reigning Schah of Persia made him a present of a small quantity of this precious bitumen in a golden box, as an object to which he attached a great value.

A practice, therefore, which we may suppose to have been confined, in the first instance, to the first classes of citizens in Egypt, as a funeral luxury, to which the lower orders of people could not aspire, from the excessive costliness of the materials, may at last have descended to them when common bitumens and resins were ascertained to answer the purpose of preserving bodies from putrefaction fully as well as the more precious *mummi-aï*. Hence the practice became general; and to that circumstance may we ascribe the large number of preparations of that kind found at Thebes, and in the immense catacombs of Saqqârah in Lower Egypt.

My only object, in giving the description of another mummy on the present occasion, is to dwell on a few points connected with one or two questions of professional importance.

The first relates to the art of bandaging different parts of the human body, as displayed in the best sort of Egyptian mummies. That which I opened was acknowledged, by the medical gentlemen present, to offer a model of the art in question, scarcely equalled, and certainly not to be surpassed by the most dexterous of our modern surgeons. To judge of the model before us, not only must the art of applying bandages be of very ancient origin, (since the mummy itself is, from accurate calculation, upwards of three thousand years old,) but it would appear that no improvement of any importance has been made in that art in subsequent ages. In the progress of unfolding the various bands by which the body of the mummy, the head, the arms, and lower extremities, in the present instance, were encircled, we recognized almost every species of bandage described in books of surgery, very neatly and artfully employed. The *circular*, the *spiral*, the *uniting*, the *retaining*, the *expellent*, and the *creeping*, were each in succession discovered in some part of the body. The *couvrechef* was not forgotten, nor the simple split-cloth, or *linteum scissum*, and the *capistrum*. But by far the neatest application of all was that of the common roller to the legs and arms, with the appropriate use of compresses to fill up hollows and depressions in the limbs. The turns laid over one another by just one half of the breadth of the bandage, and not a single reverse or *renversée*, which modern surgeons deem indispensable to the even application of this useful bandage, occurred in any part of it; yet were the parts as smooth as possible, without a wrinkle or any appearance of slackness from the varying form of the limb. There is another remark I wish to make on this subject, respecting the materials employed for swathing the mummy. The principal rollers appeared to be made of a very compact, yet elastic linen, some of them of great length, (from four to five yards,) without any seam or a single stitch appearing in any part of them. Other lesser bandages, and particularly the square envellopes of the abdomen, thorax, and head, are of cotton, and of a less elastic texture. The observation, therefore, of some recent writers, that the employment of cotton is a more recent method, is not correct. The square cloths, in the present instance, were found to alternate with the complete swathing of the whole body. They occurred four distinct times; while the bandaging, with rollers and split linen, &c. was repeated at least twenty times; the five superior or external layers being so contrived that the shape, form, and position of the limbs, lie completely concealed, and the mummy presents a homogeneous mass, with the outline, thus—

I caused the whole of the materials employed in these different sort of bandages to be weighed, and found them to be twenty-eight pounds avoirdupois. The whole of the linen employed seemed highly impregnated with the same bituminous matter with which the body itself was embalmed; and, from some experiments made since, I judge it to have been steeped into a strong solution of tannin. This is a curious fact, which, I imagine, has not yet been noticed.

Having removed, after a very laborious operation of upwards of an hour, the "outward trappings" of the mummy, the next points, connected with questions of a professional nature, for us to consider, was the sex of the individual, its state of integrity, and the mode in which the viscera had been removed.—A detail of each of these points I must defer till the next Number, as they involve matter of too much interest to admit of being briefly considered within the short space which the present Number affords me for that purpose.—A. B. G.

## Figures and Tables

**Figure f1:**