# Errata in Last Number

**Published:** 1821-11

**Authors:** 


					\oi !ne *ro,n Hie bottom, for into read in.

				

